# Intestinal helminth co-infection is an unrecognised risk factor for increased pneumococcal carriage density and invasive disease

**DOI:** 10.1038/s41598-021-86508-4

**Published:** 2021-03-26

**Authors:** Alice E. Law, Rebecca K. Shears, Andrea A. Lopez Rodas, Richard K. Grencis, Philip J. Cooper, Daniel R. Neill, Aras Kadioglu

**Affiliations:** 1 Department of Clinical Immunology, Microbiology and Immunology, Institute of Infection, Veterinary and Ecological Sciences, University of Liverpool, Liverpool, UK; 2 School of Medicine, Universidad Internacional del Ecuador, Quito, Ecuador; 3 School of Biological Sciences, Lydia Becker Institute of Immunology and Inflammation, Wellcome Centre for Cell Matrix Research, Faculty of Biology, Medicine and Health, Manchester Academic Health Science Centre, University of Manchester, Manchester, UK; 4 Institute of Infection and Immunity, St George’s University of London, London, UK

**Keywords:** Microbiology, Bacteria, Clinical microbiology, Parasitology, Infection, Infectious diseases

## Abstract

Infection with *Streptococcus pneumoniae* is the leading cause of death in children and burden of disease is greatest where helminth infections are also common. We investigated the impact of intestinal helminth co-infection on pneumococcal carriage; a risk factor for invasive disease. We used a mouse co-infection model and clinical data to assess the impact of co-infection on carriage density. Co-infection in mice was associated with increased pneumococcal carriage density and dissemination into lungs. Helminth-infected children also exhibited increased carriage density as compared to uninfected children. Anthelmintic treatment may be a cost-effective method of reducing pneumococcal disease burden in lower-income countries.

## Introduction

Multi-species co-infections pose one of the greatest challenges to world health, particularly in lower-income countries where major global pathogens such as HIV, malaria and soil-transmitted helminth (STH) infection are co-endemic^1^. A pathogen’s disease potential can be magnified by their relationship with other pathogens and co-infections involving intestinal helminths are of particular interest due to their ability to modulate systemic host immunity^2–4^. This modulation ensures chronicity of infection from childhood through to adult life and influences immune homeostasis and inflammatory responses to microbial pathogens^3,4^.

*Streptococcus pneumoniae* is the leading bacterial cause of child mortality, accounting for 341,000 deaths in children under-5 per annum^5^. The spectrum of pneumococcal diseases ranges from localised infections, such as sinusitis or otitis media, through to pneumonia, sepsis and meningitis^6^. Colonisation of the human nasopharynx by the pneumococcus is known to be a prerequisite for invasive disease^7^. Where carriage rates are high, pneumococcal pneumonia, and other forms of invasive pneumococcal disease, are a significant burden to public health services^7^ and this is most apparent within resource-constrained regions of the world^6^. STH infections are prevalent amongst these same populations, with an estimated 1.45 billion individuals infected worldwide^8^. The main species of STHs of clinical relevance are *Trichuris trichiura, Ascaris lumbricoides* and hookworms^8^. Most infants acquire pneumococcal carriage early in life, with numerous independent colonisation events occurring throughout childhood^7^. Early childhood is a time of exposure to multiple pathogens, including STHs^8^, and so co-infection is likely a common occurrence. However, detailed studies to define the incidence of pneumococcal-STH co-infection in human populations have yet to be performed.

## Results

### Intestinal helminth co-infection increases pneumococcal carriage density and leads to significantly enhanced dissemination into lungs

**Figure 1:**
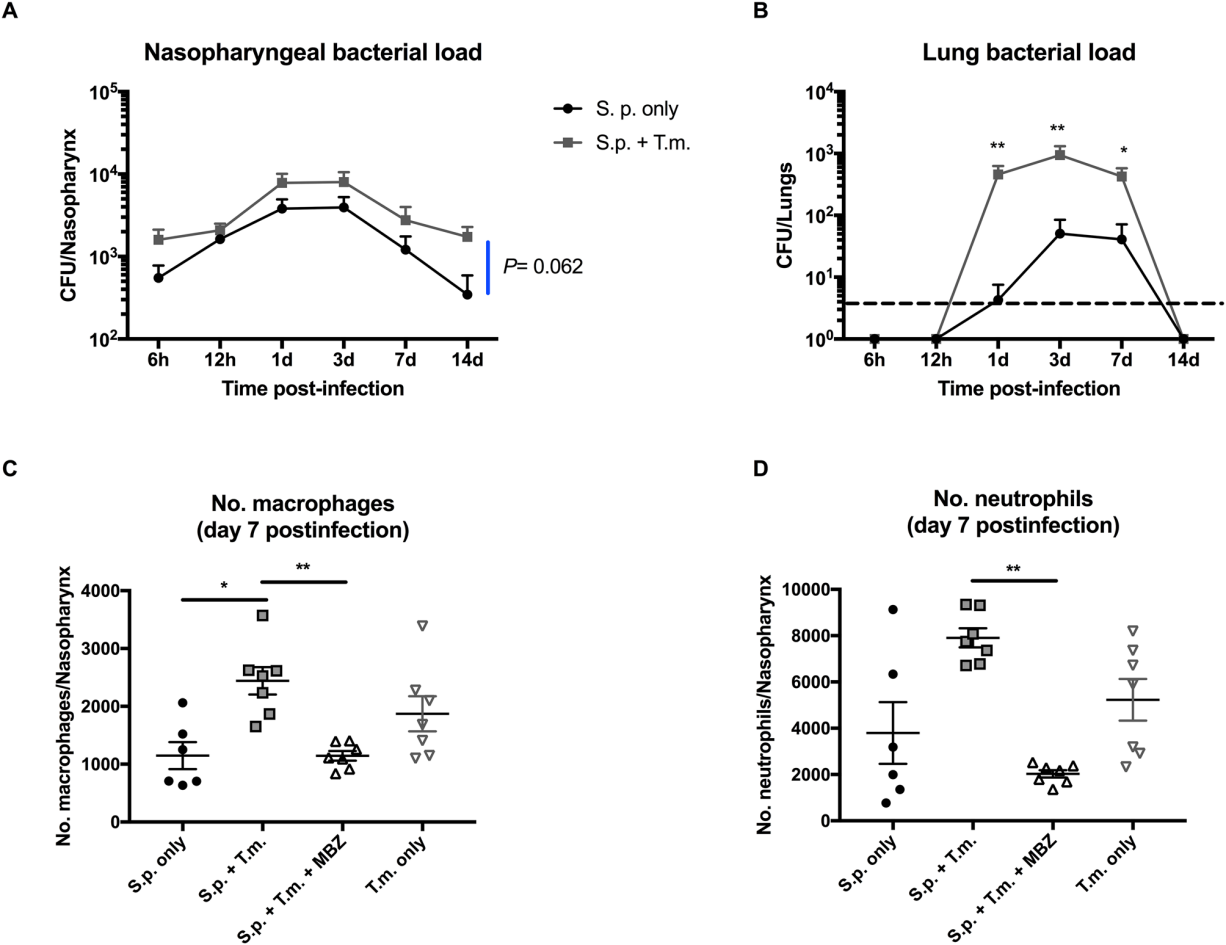
Effects of intestinal helminth co-infection and anthelmintic treatment on pneumococcal infection dynamics and host immune responses. Mice were infected with 20 T*. muris* eggs by oral gavage, followed by induction of pneumococcal carriage 28 days later. Co-infected mice had a trend towards increased nasopharyngeal carriage density as compared to pneumococcus only infected animals (**A**) and this was accompanied by significantly increased pneumococcal dissemination into lungs of co-infected animals (**B**). Co-infection was accompanied by increased macrophages (**C**) and neutrophils (**D**), which was reversed by anthelminthic treatment. *S.p.* = *S. pneumoniae, T.m.* = *T. muris,* MBZ = mebendazole, 6 h = 6 hours, 1d = 1 day. Mean and SEM are indicated, *(*P* < 0.05) and **(*P* < 0.01). Broken line indicates limit of detection (50 colonies).

We established a *T. muris* (mouse whipworm) and *S. pneumoniae* co-infection model to determine whether STH infection altered pneumococcal carriage density, and/or dissemination into the lower airways. Mice were infected with *T. muris*, and 28 days later, when the worms had matured into adults, mice were infected with a carriage-inducing dose of *S. pneumoniae*. Co-infection with *T. muris* led to a consistent increase in nasopharyngeal carriage density over time, which may be biologically important, although this did not reach statistical significance (*P* = 0.062, Fig. 1A). In the lungs, a substantial difference was observed whereby co-infected mice had significantly increased pneumococcal dissemination into their lungs at 1, 3, and 7 days post-infection (Fig. 1B). Co-infection led to a significant increase in the number of macrophages within the nasopharynx compared to mono-infected animals (*P* = 0.0118, Fig. 1C) and a clear trend towards increased neutrophils (*P* = 0.0561, Fig. 1D), which was reversed by anti-helminthic treatment (*P* = 0.0083 and 0.0019 for macrophages and neutrophils respectively). Anthelminthic treatment also led to a small (but non-significant, *P* = 0.075) reduction in nasopharyngeal bacterial load (Fig. S1).

### Intestinal helminth co-infection is a risk factor for increased pneumococcal carriage density in Ecuadorian children

**Figure 2:**
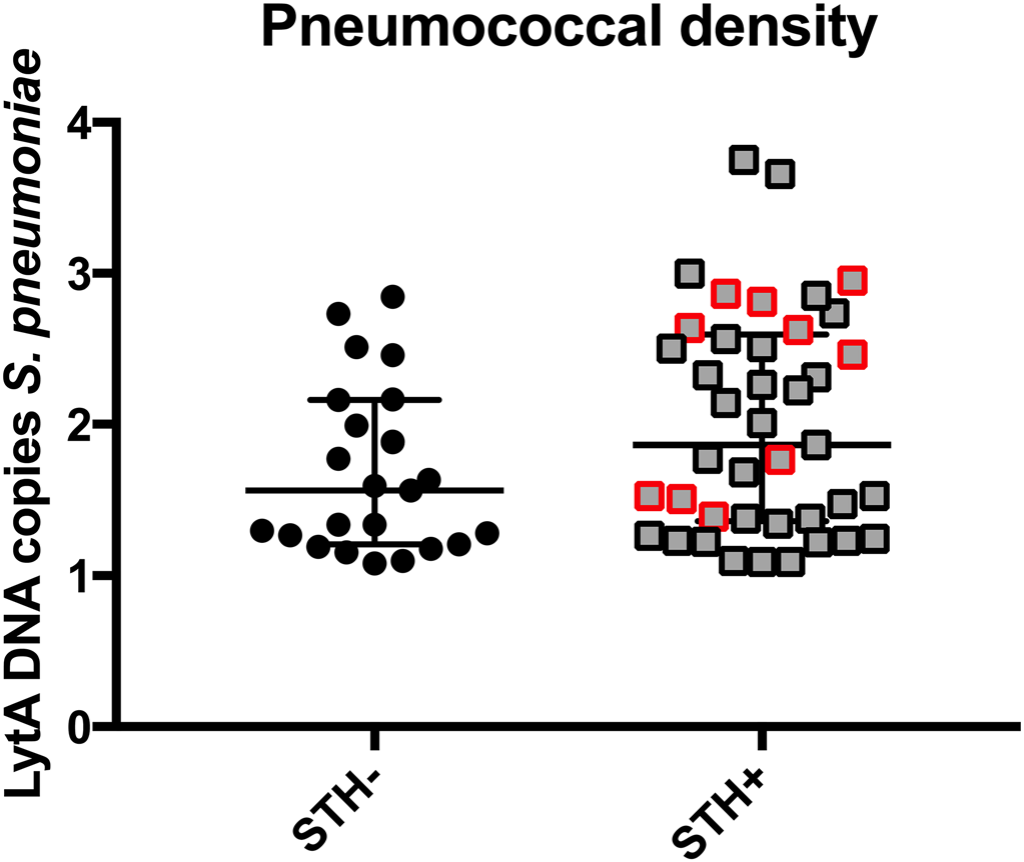
Pneumococcal carriage density in STH-uninfected versus STH-infected children. *lytA* PCR was performed on oropharyngeal swabs collected as part of the ECUAVIDA cohort study in order to determine pneumococcal carriage density in STH-uninfected (STH-) and STH-infected (STH +) respectively. STH + includes *T. trichiura, A. lumbricoides* and mixed *T. trichiura/A. lumbricoides* infections combined. Red outline indicates *A. lumbricoides* infections alone. Median and IQR are indicated.

We next investigated whether STH co-infection was also associated with increased pneumococcal carriage density in children. A total of 387 children enrolled in the ECUAVIDA study (age range 54–95 months, median = 60 months) were tested for pneumococcal carriage as measured by *lytA* PCR performed on oropharyngeal swabs, collected as described^9^. Pneumococcal carriage density (as measured by number of *lytA* copies) was higher in STH-infected children than uninfected children (Fig. 2), although this did not reach significance. *A. lumbricoides* infections were associated with the largest increase in pneumococcal density (*P* = 0.029, univariable analysis) of all the factors measured (Table 1). Multivariable analysis was performed with all helminth infections and age, sex and household overcrowding were included a priori^10^. PCV13 vaccination status was not included in the analyses, as only 1.8% of children sampled had received the vaccine.

**Table 1:** Linear regression analysis of factors associated with *S. pneumoniae* density in children aged 54–95 months who are carriers.

Variable	Summary statistic Carriers/ total (%)	Log10 LytA DNA copies/mL	Univariable analysis		Multivariable analysis	
			Crude coefficient^a^ (95% CI)	P value	Adjusted coefficient^a^ (95% CI)	P value
Age (n = 387)	68/387 (17.5)	1.9	− 0.412 (− 0.06, 0.04)	0.682	− 0.024 (− 0.06, 0.05)	0.847
**Sex (n = 391)**				0.18		0.367
Male	38/204 (18.6)	1.79	1			
Female	30/187 (16.0)	2.03	0.170 (− 0.11, 0.58)		0.118 (− 0.19, 0.51)	
**Socioeconomic status (n = 231)**^b^						
Medium	13/100 (13.0)	2.06	1			
High	26/131 (19.7)	1.98	− 0.06 (− 0.38, 0.55)	0.722		
**Household (n = 391)**						
Urban	47/259 (18.1)	1.95	1			
Rural	21/132 (15.9)	1.76	− 0.127 (− 0.56, 0.19)	0.319		
**Household overcrowding (n = 391)**						0.077
No	30/189 (15.9)	2.05	1			
Yes	38/202 (18.8)	1.77	− 0.206 (− 0.62, 0.06)	0.103	− 0.236 (− 0.69, 0.36)	
**Respiratory symptoms (wheeze) (n = 391)**						
No	59/242 (24.4)	1.93	1			
Yes	9/49 (18.4)	1.71	− 0.108 (− 0.71, 0.28)	0.394		
**Type of helminth infection (n = 391)**						
No active infection	25/115 (21.7)	1.69	1		1	
*T. Trichuira*	14/100 (14.0)	1.94	0.156 (− 0.20, 0.71)	0.265	0.142 (− 0.25, 0.72)	0.339
*A. Lumbricoides*	11/88 (12.5)	2.26	0.304 (0.59, 1.08)	0.029	0.275 (− 0.003, 1.04)	0.051
Mixed	18/88 (20.5)	1.93	0.158 (− 0.19, 0.68)	0.263	0.214 (− 0.13, 0.81)	0.154

Analysis was restricted to positive carriers (no. of carriers/total for each category is indicated in summary statistic column) and results are reported as linear regression coefficients and 95% CI. All helminth infections alongside age and sex were then used in a multivariable regression model to estimate adjusted coefficients and identify independent associations. ^a^Coeficient was log10 converted, e.g. those with *A. lumbricoides* infection have a pneumococcal density of 0.3 log10 higher than those with no active infection. ^b^The first component that accounted for 30% of variation was divided into tertiles to represent low, middle and high socioeconomic status as described by Menzies et al*.* (2014)^22^. Median age for participants was 60 months, IQR = 1 (54–95).

## Discussion

The impact of a single pathogen is likely multiplied in the context of one or more co-infections in complex and challenging ways, particularly for STHs, which have evolved to modulate the host immune response, in order to favour chronicity of infection^4^. The impact of STH co-infection on pneumococcal disease burden and outcome is largely unknown despite sharing prevalence in the same geographical regions of the world. Therefore, we sought to study this using a combination of mouse models and human data. Here, we demonstrate that the murine intestinal helminth, *T. muris*, enhances pneumococcal (D39) nasopharyngeal carriage density and significantly increases pneumococcal CFU load in the lungs. Although D39 is virulent in mice, it does not cause invasive pneumonia when used in models of carriage, in the absence of other stimuli, instead providing a stable and reproducible model of long-term asymptomatic nasopharyngeal infection. It thus offers an opportunity to investigate bacterial, host and environmental factors which control carriage or that perturb stable nasopharyngeal infection towards invasive disease^11–13^. We show that co-infection with *T. muris* drives host immune cell infiltration with increased macrophages and neutrophils in the nasopharynx of co-infected mice; suggesting a pro-inflammatory environment conducive to increased pneumococcal lung dissemination, as has been shown previously^14^. Interestingly, increases in pneumococcal carriage density and cellular infiltration were both reduced by anthelmintic treatment. Similar mechanisms may also be at play in humans, as we observed increased pneumococcal carriage density in helminth-infected Ecuadorian children, as compared to uninfected children, and previous studies have shown a link between increased carriage density and pneumococcal pneumonia in both mice and humans^11,14–17^. As the *lytA* qPCR is not as specific for oropharyngeal swabs as it is for nasopharyngeal swabs, we sought to confirm pneumococci with a second PCR for *cpsA*; there was a positive correlation between pneumococcal DNA copies of both genes as determined by cpsA and lytA qPCR (P < 0.0001). The largest difference in pneumococcal density was recorded in *A. lumbricoides* infected children, however, it is not possible to study chronic *A. lumbricoides* infection in mice since the parasite is expelled before the adult worms establish in the gastrointestinal tract^18^, which is why we opted for another roundworm species, *T. muris*, a close relative of *T. trichuira*^19^. Our study had a relatively small sample size (68 *lytA* positive children out of 387 in total), and therefore it is possible that interactions may have been missed where effect sizes were small, for instance in the case of *T. trichuira* co-infection. Similarly, some of the observations reported using our mouse model fall short of reaching statistical significance (for example, the increase in nasopharyngeal neutrophil count for co-infected versus those infected with pneumococci only). Future studies should seek to further validate these observations as well as providing additional mechanistic understanding of these processes.

Overall, these data suggest that coordinated deworming programmes alongside pneumococcal vaccination may aid in the reduction of pneumococcal disease in areas of the world where STH infections are also common. Pneumococcal carriage is known to be a risk factor for pneumococcal pneumonia, and non-vaccine therapeutic methods to reduce carriage density could also have a major impact on the prevalence of pneumococcal pneumonia at low economic cost^7^.

In summary, our data suggest that intestinal helminth co-infection is associated with increased levels of pneumococcal carriage and a clear and significant increase in bacterial dissemination to the lower airways. Given that the incidence of pneumococcal disease is highest in areas of the world where STH infections are most common, these data suggest that anthelminthic treatment could be a cost-effective method to reduce pneumococcal disease in lower-income countries.

## Methods

All experimental protocols were approved by either the University of Liverpool Ethical and Animal Welfare Committee or by the ethics committees of the Hospital Pedro Vicente Maldonado and the Universidad San Francisco de Quito.

### Mice

All experimental protocols were performed in accordance with the Home Office Scientific Procedures Act (1986), Project Licence P86De83DA, and the University of Liverpool Ethical and Animal Welfare Committee, which approved the study. All procedures were carried out in compliance with ARRIVE guidelines. All procedures were carried out on MF1 mice 6–8 weeks or older. Mice were acclimatised for one-week prior to use and had free access to food and water in individually-ventilated cages.

### Bacteria

*S. pneumoniae* Serotype 2 strain D39 (NCTC 7466) was used for all in vivo experiments. Bacteria were cultured as described previously^11^.

### Parasites

The Edinburgh (E) strain of *T.* *muris* was used for all experiments. Parasite maintenance was carried out at the University of Manchester, as described previously^20^.

### Mouse model of pneumococcal/helminth co-infection

Mice were infected by oral gavage with 20 T*. muris* embryonated eggs in 200µL ddH_2_O. Control mice were sham infected with 200µL ddH_2_O. Twenty-eight days later, mice were lightly anaesthetised with a mixture of O_2_ and isofluorane and infected intranasally with 1 × 10^5^ colony-forming units (CFU) *S. pneumoniae* D39 in 10µL PBS, as previously described^11^. In those animals treated with the anthelmintic, mebendazole, the drug was administered by oral gavage once daily (100 mg/kg) for three days, immediately after *S. pneumoniae* infection. Control mice (S.p. only and S.p. + T.m. groups) were sham treated with 200µL PBS by oral gavage. At pre-selected time intervals following intranasal infection, mice were humanely culled, nasopharynx and lungs were removed and blood was collected via cardiac puncture. A viable count of bacteria was determined at each interval by serial dilution of tissue homogenates onto blood agar, as previously described^11^. Worm burden was measured as described previously^20^. Single cell suspension from the nasopharynx were stained as follows: CD45-FITC, GR1-PE-CY7 (RB6-8C5, BD Biosciences), CD11b-PE (M1/70, Biolegend) and F4/80-Pacific blue (T45-2342, BD Biosciences). Acquisition was carried out using a FACSCanto flow cytometer (BD Biosciences) and analysis performed using Flowjo X (Treestar). Fluorescent minus one controls for each of the included antibodies were used to validate results.

### Assessment of rate and density of *S. pneumoniae* carriage in Ecuadorian children

Total DNA was extracted from oropharyngeal swabs collected as part of the ECUAVIDA cohort study^9^ using an Agowa Mag mini DNA extraction kit (LGC Genomics). The ECUAVIDA cohort consists of 2404 newborns recruited between 2006 and 2009 to study the impact of soil-transmitted helminths (STHs) and early-life microbial exposures on the development of atopy, allergic diseases and immune responses in childhood. Informed written consent was obtained from the mothers of participating children and all methods were carried out in accordance with local guidelines and regulations^9^. The ECUAVIDA study was approved by the Ministry of Health in Ecuador and by the ethics committees of the Hospital Pedro Vicente Maldonado and the Universidad San Francisco de Quito. The study is registered as an observational study (ISRCTN 41,239,086). Carriage density by qPCR was determined by partial amplification of the *lytA* gene of *S.* *pneumoniae*, as described previously^21^. Carriage density was also assessed using the *cspA* gene and there was a positive correlation between pneumococcal DNA copies of both genes as determined by *cpsA* and *lytA* qPCR (*P* < 0.0001, Fig. S2). Samples were only classified as positive for pneumococci if both *lytA* and *cpsA* qPCR signals were > 10 DNA copies (< 40 cycles). 49 swabs were determined to be negative for pneumococci based upon dissimilarity between the *lytA* and *cpsA* qPCR results. Overall, 68 swabs were determined as pneumococci positive. Anthelmintic treatment was administered to children who tested positive for helminth infection for ethical reasons.

### Statistical analysis

For the murine experiments, statistical analysis was carried out using GraphPad Prism 7. Mann–Whitney U test and ANOVA with Kruskall-Wallis post-test were performed when comparing two and three or more experimental groups, respectively. Error bars represent SEM. Statistical significance is denoted by *(*P* < 0.05) and **(*P* < 0.01).

For clinical data, statistical analysis was performed using IBM SPSS version 25. Bacterial density data were log_10_ transformed and linear regression was used to assess relationships between potential risk factors and colonisation density. Analysis was restricted to positive carriers and results were reported as linear regression coefficients and 95% confidence interval (CI). Covariates significant at *P* < 0.1 alongside age, sex and household crowding (selected a priori)^10^ were then used in a multivariable regression model to estimate adjusted coefficients and identify independent associations.

## Supplementary Information

Supplementary Information
