# Supplementary material for: Intestinal helminth co-infection is an unrecognised risk factor for increased pneumococcal carriage density and invasive disease
Source: Sci Rep. 2021 Mar 26;11:6984. doi: 10.1038/s41598-021-86508-4 (PMC7997997; doi:10.1038/s41598-021-86508-4)

**Intestinal helminth co-infection is an unrecognised risk factor for increased pneumococcal carriage density and invasive disease.**

Alice E. Law^1†^, Rebecca K. Shears^1†^, Andrea A. Lopez Rodas^2^, Richard K. Grencis^3^, Philip J. Cooper^2,4^, Daniel R. Neill^1*^, Aras Kadioglu^1*^**^¶^**

^1^Department of Clinical Immunology, Microbiology and Immunology, Institute of Infection, Veterinary and Ecological Sciences, University of Liverpool, Liverpool, UK

^2^School of Medicine, Universidad Internacional del Ecuador, Quito, Ecuador

^3^School of Biological Sciences, Lydia Becker Institute of Immunology and Inflammation, Wellcome Centre for Cell Matrix Research, Faculty of Biology, Medicine and Health, Manchester Academic Health Science Centre, University of Manchester, Manchester, UK

^4^Institute of Infection and Immunity, St George’s University of London

^†^These authors contributed equally

^*^Co-senior authors

**^¶^** Correspondence to:

Prof. Aras Kadioglu

Department of Clinical Immunology, Microbiology and Immunology, Institute of Infection and Global Health, University of Liverpool, Liverpool, UK

a.kadioglu@liverpool.ac.uk

**Key words:** pneumococcus, nasopharyngeal carriage, invasive pneumococcal disease, co-infection, helminths.

**Fig S1. Anthelminthic (mebendazole) treatment led to a small reduction in nasopharyngeal bacterial load.** *S.p.*= *S. pneumoniae, T.m.= T. muris,* MBZ= mebendazole. Mean and SEM are indicated.


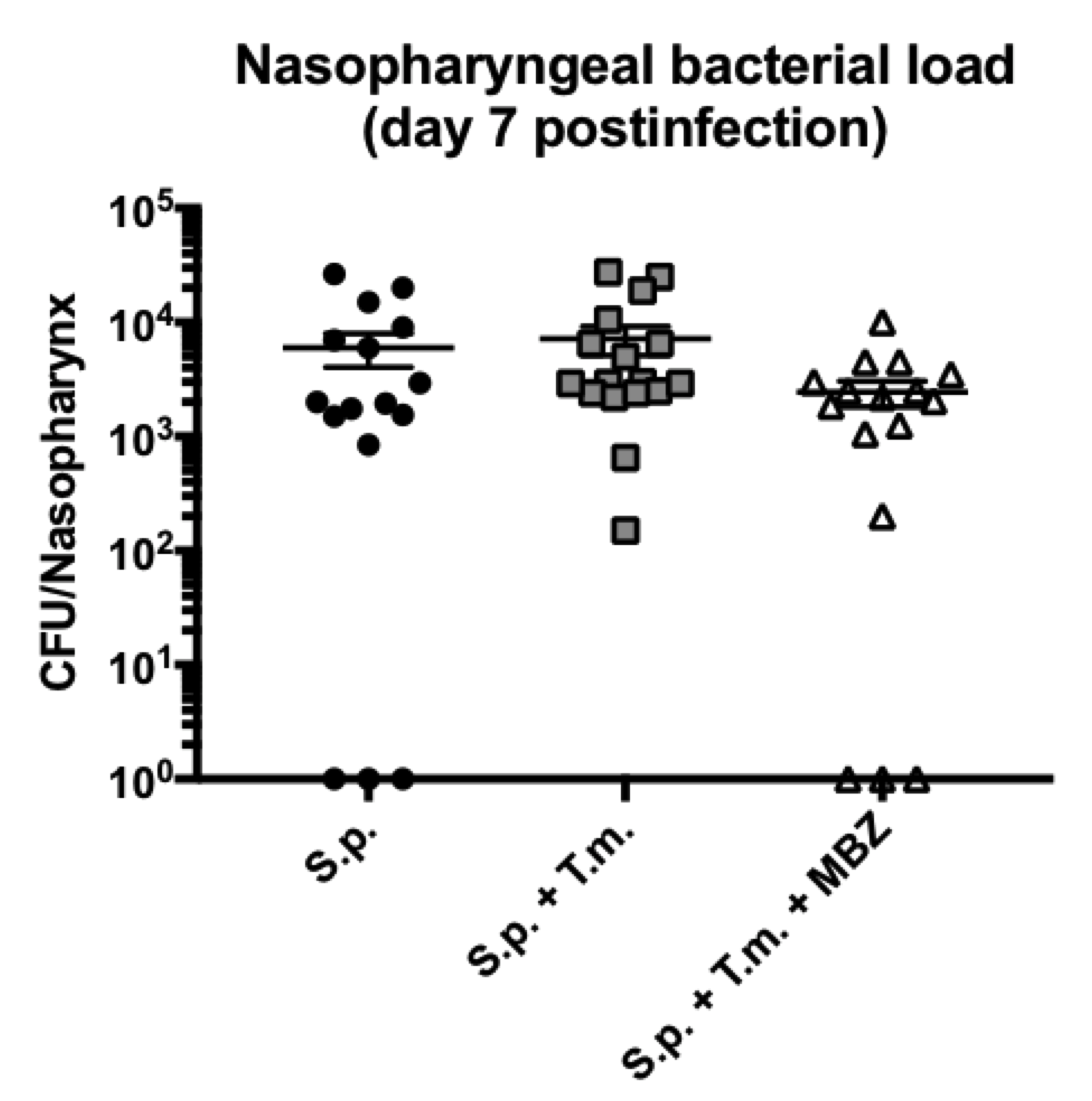


**Fig S2. Correlation between *lytA* and *cpsA* DNA copies.** To address concerns of the specificity, oropharyngeal pneumococcal carriage density was assessed via qPCR targeting both *lytA* and the *cpsA* genes. There was a positive correlation between pneumococcal DNA copies of both genes as determined by *cpsA* and *lytA* qPCR (*P* < 0.0001).


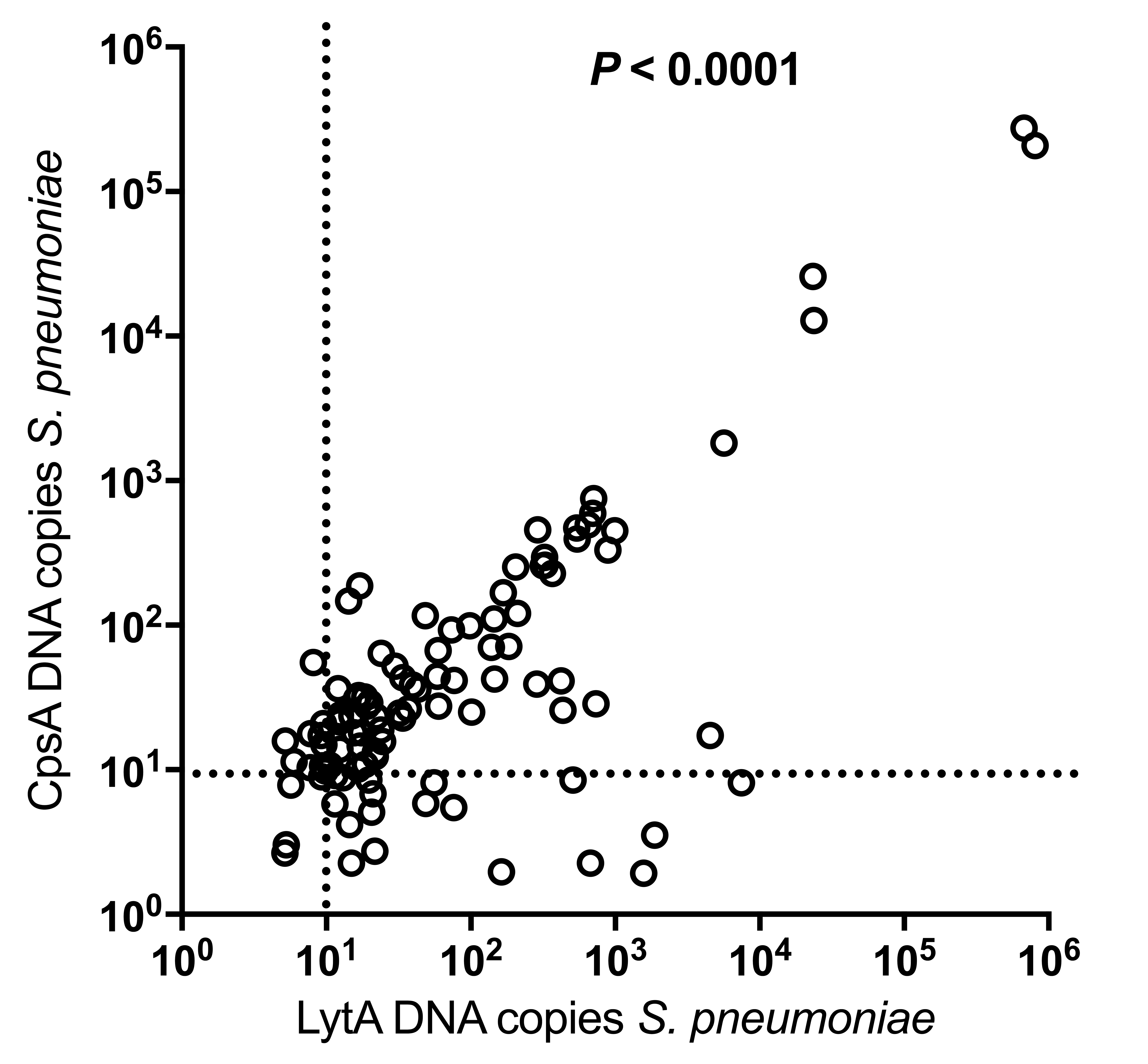

Supplement: Supplementary file 1 — Supplementary Information [file 41598_2021_86508_MOESM1_ESM.docx]
